# Discovery of PTN as a serum-based biomarker of pro-metastatic prostate cancer

**DOI:** 10.1038/s41416-020-01200-0

**Published:** 2020-12-08

**Authors:** Shiqin Liu, Michelle Shen, En-Chi Hsu, Chiyuan Amy Zhang, Fernando Garcia-Marques, Rosalie Nolley, Kashyap Koul, Meghan A. Rice, Merve Aslan, Sharon J. Pitteri, Charlie Massie, Anne George, James D. Brooks, Vincent J. Gnanapragasam, Tanya Stoyanova

**Affiliations:** 1 Department of Radiology, Stanford University, Stanford, CA USA; 2 Canary Center at Stanford for Cancer Early Detection, Stanford University, Palo Alto, CA USA; 3 Department of Urology, Stanford University, Stanford, CA USA; 4 Cambridge Urology Translational Research and Clinical Trials, Cambridge University Hospitals NHS Trust & University of Cambridge, Cambridge, UK; 5 Urological Malignancies Programme, CRUK Cambridge Cancer Centre, Cambridge, UK; 6 Early Detection Programme, CRUK Cambridge Cancer Centre, Cambridge, UK; 7 Academic Urology Group, Department of Surgery, University of Cambridge, Cambridge, UK

**Keywords:** Prostate cancer, Cancer screening

## Abstract

Distinguishing clinically significant from indolent prostate cancer (PC) is a major clinical challenge. We utilised targeted protein biomarker discovery approach to identify biomarkers specific for pro-metastatic PC. Serum samples from the cancer-free group; Cambridge Prognostic Group 1 (CPG1, low risk); CPG5 (high risk) and metastatic disease were analysed using Olink Proteomics panels. Tissue validation was performed by immunohistochemistry in a radical prostatectomy cohort (*n* = 234). We discovered that nine proteins (pleiotrophin (PTN), MK, PVRL4, EPHA2, TFPI-2, hK11, SYND1, ANGPT2, and hK14) were elevated in metastatic PC patients when compared to other groups. PTN levels were increased in serum from men with CPG5 compared to benign and CPG1. High tissue PTN level was an independent predictor of biochemical recurrence and metastatic progression in low- and intermediate-grade disease. These findings suggest that PTN may represent a novel biomarker for the presence of poor prognosis local disease with the potential to metastasise warranting further investigation.

## Background

Prostate cancer (PC) is the most common non-cutaneous cancer and the second leading cause of cancer-associated deaths among men.^1^ Since its discovery, prostate-specific antigen (PSA) remains the most widely used serum-based biomarker for PC prognostic assessment and treatment decisions. However, PSA cannot distinguish between low-risk patients who do not need treatment and clinically significant disease, which will have a poor prognosis if not treated.^2^ Many screening studies using PSA have shown that while more cancers can be found, many of these are indolent and are overtreated. As such, the use of PSA as a screening test remains highly controversial.^3^ Instead, the focus has shifted to the development of biomarkers for clinically significant disease. Significant progress has been made in developing new and improved biomarkers such as the prostate health index, and the STHLM3 test.^4^ Urine biomarkers such as PCA3 and Select Mdx have also shown the potential to improve PC detection specificity. Although significant improvements over PSA alone, these tests still identify many cancers that may not cause harm if un-detected. Recent work from our group and others have demonstrated that many men with intermediate-risk disease will do very well from a non-intervention approach.^5,6^ The majority of deaths from PC are also known to occur in men who present with already metastatic disease.^7^ Therefore, to really have an impact on reducing mortality while also reducing over-detection, a biomarker should be able to detect cancers with the hallmarks of future metastatic potential. The goal of this work was to identify proteins that could predict metastatic potential in serum samples taken at diagnosis from men with good and poor prognosis non-metastatic PC as well as established disseminated disease.

## Methods

### Patient serum and tumour tissue samples

#### The Cambridge serum cohort

The Cambridge Prognostic Groups define five prognostic stratifications for men with non-metastatic disease, each with increasing risks of disease-specific mortality (CPG1–5). The prognostic power of this classification system has been independently validated. Men in CPG1 have a >97% 10-year disease-specific survival, whereas men with CPG5 disease have a 50% 10-year survival. The CPGs are routinely used to classify all men recruited into the Cambridge Urological Biorepository (DIAMOND study CI VJG, Ethics 03/018). Patient serums from 80 men recruited at the point of diagnosis from this repository were utilised as our discovery cohort. Of these, 20 men confirmed the absence of PC; 20 men were in CPG1; 20 men were in CPG5, and 20 men presented with established metastatic disease (Supplementary Table S1). All men were diagnosed through the use of image-guided biopsies with pre-biopsy magnetic resonance imaging and a combination of targeted and systematic transrectal or transperineal biopsies. Metastasis was defined as a positive bone scan and/or lymph nodes.

#### The Stanford tissue microarray (TMA) cohort

The samples used to build the Stanford TMAs have been collected under IRB-approved protocols from over 600 men. Diagnosed with PC between 1996 and 2009, these patients were treated with radical prostatectomy at the Stanford University. The database contains their deidentified clinical information, including pathology reports, two-dimensional tumour measurements at the greatest diameter (a validated surrogate of tumour volume), patient age, pre-operative PSA levels, clinical stage, pathological data on capsular penetration, seminal vesicle invasion, lymph node invasion, and positive surgical margins. Clinical follow-up information, such as post-operative PSA, post-operative imaging and subsequent treatments for patients with detectable PSA, is also present in this database. The TMAs contain samples from 234 patients with four tissue core biopsies per patient with a median follow-up of 10 years.

### Targeted proteomics

**Fig. 1:**
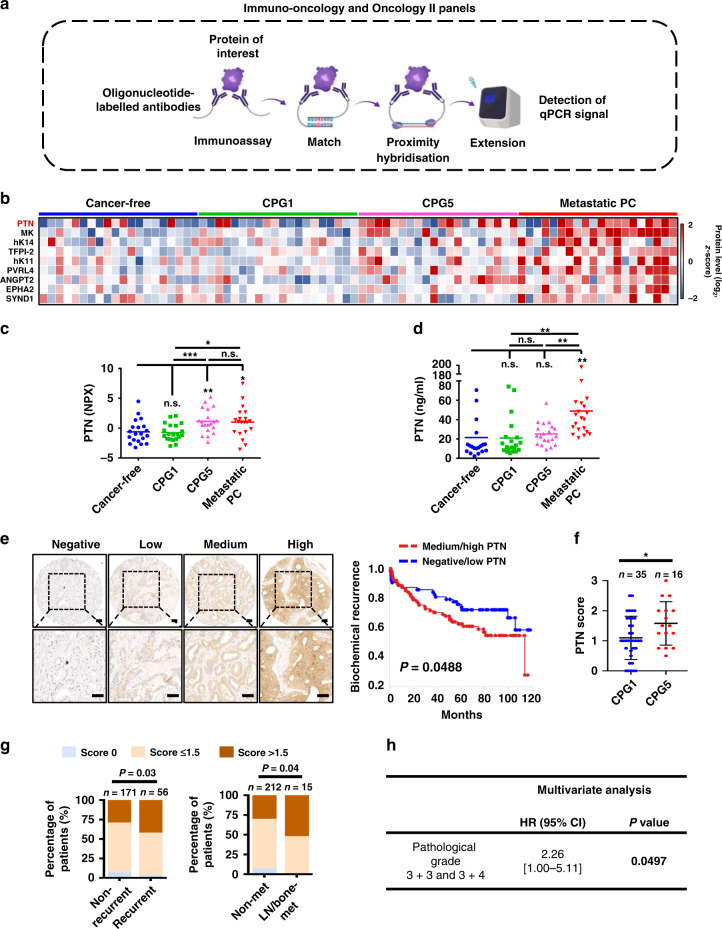
Discovery of PTN as a potential serum-based biomarker for clinically significant prostate cancer through high-multiplex immunoassays. **a** Schematic representation of Olink targeted proteomics method. Immuno-Oncology and Oncology II panels utilise proximity extension assay (PEA) to measure 92 human proteins associated with immuno-oncology and 92 human protein biomarkers related to oncology. The detection of each protein is achieved by paired oligonucleotide-labelled antibodies. Binding of the antibodies to the protein target leads to DNA hybridisation upon proximity, resulting in the formation of a DNA reporter sequence. The DNA reporter sequence is quantified by qPCR. A list of the proteins in each panel is shown in Supplementary Table S2. The illustration was generated using BioRender (https://biorender.com). **b** Eighty serum samples from four different patient groups: (1) cancer-free group, (2) the Cambridge Prognostic Group 1 (CPG1), disease with 97% 10-year survival, (3) CPG5, disease with 50% 10-year survival and (4) men with metastatic disease since diagnosis were analysed using Immuno-Oncology and Oncology II panels. *N* = 20 per group. Heatmap of nine proteins found to be elevated in at least one group in the proteomics. Protein level (log_2_, *z*-score). **c** Normalised protein expression (NPX) for pleiotrophin (PTN) is plotted. **d** PTN levels in the 80 serum samples used for proteomics analysis were analysed by Sandwich ELISA. Error bars represent standard deviation (SD). For all, *P < 0.05, **P < 0.01, ***P < 0.005, ****P < 0.001 and n.s. not significant. Significance was determined by Student’s *t* test (two-tailed). **e** IHC staining of PTN on Stanford University tissue microarrays (TMAs). PTN staining intensity was scored from 0 to 3 (0 is negative, 1 is low, 2 is medium, 3 is high). Representative images for negative, low, medium and high PTN staining are shown in the right panel. Scale bars represent 50 μm (upper panel) and 25 μm (lower panel), respectively. PTN IHC on Stanford University TMAs with associated clinical data is shown in the left panel. High PTN expression correlates with lower 10-year recurrence-free survival. *P* = 0.049. **f** Comparative PTN IHC scores between those with CPG1 and CPG5 disease. **g** The distribution of PTN intensity scores as a percentage of patient samples. PTN score 0: PTN score of four different cores = 0; PTN score ≤ 1.5: average PTN score of four different cores ≤ 1.5; PTN score > 1.5: average PTN score of four different cores > 1.5. Strong staining for PTN correlates with prostate cancer recurrence after prostatectomy (recurrent patients: *n* = 56; non-recurrent patients: *n* = 171) (left panel). The statistical significance of the differences between non-recurrent and recurrent proportions was calculated by *z*-score normal distribution *N* (0,1). *P* = 0.03. High PTN levels correlate with prostate cancer metastasis (right panel). Samples from patients with no metastasis (*n* = 212) and patients with metastasis (*n* = 15, including nine patients with lymph node metastasis, three patients with bone metastasis, and three patients with both lymph node and bone metastasis) were analysed. The statistical significance of the differences between no metastasis and metastasis proportions was calculated by the normal distribution *N* (0,1) of *z*-scores. *P* = 0.04. **h** Multivariate analysis of high levels of PTN as an independent predictor of biochemical recurrence in patients with pathological Gleason grades 3 + 3 and 3 + 4. *P* < 0.05.

The Olink Immuno-oncology and Oncology II panels utilise proximity extension assay (PEA) technology to measure the presence of 92 human proteins associated with immuno-oncology and 92 human protein biomarkers related to oncology listed in Supplementary Table S2. A pair of oligonucleotide-labelled antibodies was used to recognise and bind to each target protein. Once the two antibodies bind to the protein target, the complementary oligonucleotides attached to each antibody hybridise and a DNA reporter sequence is formed. High-throughput, real-time quantitative polymerase chain reaction is used to quantify the DNA reporter sequences (Fig. 1a).

### Sandwich enzyme-linked immunosorbent assay (ELISA)

Pleiotrophin (PTN) levels in 80 serum samples from four different patient groups (cancer-free, CPG1 and CPG5, and patients with metastatic PC) were determined by Sandwich ELISA. Diluted in 0.2 M sodium bicarbonate (pH 9.4), commercial PTN capture antibody (sc-74443, 1:250, Santa Cruz) was coated in 96-well, half-area plates (#3690, Corning) overnight at 4 °C. Then, the plates were washed three times with phosphate-buffered saline (PBS) and blocked with 5% bovine serum albumin in PBS overnight at 4 °C. Serum samples (4 μL) were incubated at room temperature for 2 h. Plates were then washed three times with PBS containing 0.01% Tween-20 (PBST) and were then incubated with biotinylated PTN detection antibody (BAF252, 1:100, R&D Systems) at room temperature for 2 h. Plates were washed three times with PBST, incubated with peroxidase-conjugated streptavidin biotin (PI21134, 1:200, Thermo Scientific) at room temperature for 1 h, washed three times with PBST and washed another two times with PBS. Bound proteins were detected using ultra TMB-ELISA substrate (#34028, as per the manufacturer’s directions, Thermo Scientific) and analysed by a Promega plate reader at 450 nm. The standard curve for PTN concentrations was determined using 0.1–200 ng/ml recombinant human PTN (252-PL-050, R&D Systems).

### Immunohistochemistry (IHC)

IHC was performed on a 4-μm section of the Stanford TMAs described above using a commercial PTN biotinylated antibody (BAF252, 1:100, R&D System). Briefly, the TMAs slides were deparaffinised at 64 °C for 1 h and then rehydrated in 100%, 95%, 90% and 70% alcohol for 5 min in each. The antigen was retrieved by using 10 mM citrate buffer (pH = 6.0) at 95 °C for 30 min. Endogenous peroxidase activity was blocked in 3% hydrogen peroxide for 5 min. Slides were blocked with 2.5% horse serum at room temperature for 1 h. PTN biotinylated antibody (1:100) was added and incubated at 4 °C overnight. The slides were washed three times with PBS and incubated with streptavidin-horseradish peroxidase (SA-5004, 1:200, Vector Laboratories) at room temperature for 1 h. The slides were washed three times in PBS, and the staining colour was detected using a DAB kit (Dako, as per the manufacturer’s protocol). The nuclei were stained with haematoxylin, and then slides were dehydrated with ascending gradient alcohol. Cancer cores were scored from 0 to 3 based on staining intensity (0 is negative, 1 is low, 2 is medium, 3 is high). If at least one of the four PTN cores had a score of 2 or 3, the patients were classified as medium/high group, while the rest if at least one of the four PTN cores had a score of 1 or 0 were classified as low/negative group. All TMA sections were scanned using a NanoZoomer (Hamamatsu).

### Statistical analysis

Summary statistics of patients’ PTN IHC score and other clinical parameters (CPG, pathological grade, pathological T stage, N stage, and surgical margin) are provided in frequencies and percentages in Supplementary Table S4. Fisher’s exact test was used to assess correlations between PTN IHC and other clinical factors. Patient age and pre-operation PSA level are summarised using the median and interquartile range. Mann–Whitney *T* test was used to compare age and pre-operative PSA levels between PTN negative/low and PTN medium/high groups. −2log (likelihood-ratio) test was used to analyse the association between PTN IHC level and biochemical recurrence. The statistical significance of the differences between non-recurrent versus recurrent and non-metastatic versus metastatic proportions was calculated through the normal distribution N (0,1) of *z*-scores. Multivariate cause-specific Cox proportional-hazards regression models were performed for the risk of biochemical recurrence. PTN expression and pathological Gleason grade were included in the analysis. All tests were two-sided, and *P* values of 0.05 or less were considered statistically significant.

## Results

In recent years, a high-multiplex immunoassay has been used to identify potential serum-based biomarkers for different types of cancers.^8^ This platform utilises a dual antibody-based recognition coupled with DNA PEA technology that enables high sensitivity and detection of multiple proteins simultaneously (Fig. 1a). We used serum samples from patients classified as benign, very low-risk, and highest risk for PC defined by a validated five-strata prognostic model using histological grade, clinical stage and PSA at diagnosis as criteria-the Cambridge Prognostic Groups (CPGs).^9,10^ Men with CPG1 disease have a greater than 97% 10-year disease-specific survival, whereas men with CPG5 disease have a 50% 10-year survival.^9^ We also included men with established metastatic disease at diagnosis. For proteomic analysis, we used patient sera from those four groups: 20 men with confirmed absence of PC by systemic and image-guided biopsy, 20 men with CPG1, 20 men with CPG5, and 20 men with metastatic disease (Supplementary Table S1). The quantitative targeted proteomics (Fig. 1a) Oncology II and Immuno-Oncology panels were used for the detection of 184 proteins (Supplementary Table S2). Proteins with <25% detectability were removed from the analysis (174 remained out of 184) (Supplementary Table S3). Nine out of 174 proteins exhibited a difference in serum levels in at least one group (Fig. 1b, c and Supplementary Fig. S1, *P* < 0.05). PTN, MK, PVRL4, EPHA2, TFPI-2, hK11, SYND1, ANGPT2 and hK14 were found elevated in the metastatic disease when compared to cancer-free, CPG1 and/or CPG5 groups (Fig. 1b, c and Supplementary Fig. S1). Levels of PTN, in particular, were also upregulated in serum from patients with CPG5 disease when compared to benign and CPG1 sera and thus was selected as a candidate biomarker for early detection of significant PC and further validation (Fig. 1c). To further validate PTN expression, a sandwich ELISA was developed using commercially available antibodies (Fig. 1d). PTN levels were again elevated in the metastatic when compared to the cancer-free, CPG1 and CPG5 patient groups consistent with the findings in the proteomic assay (Fig. 1d). We also observed a trend to higher PTN levels in CPG5 serum compared to cancer-free and CPG1 groups, although the difference was not statistically significant, which could be potentially attributed to the high sensitivity of the proteomic assay. These results demonstrate that these nine proteins may potentially represent candidate biomarkers for metastatic PC. PTN in particular may be a promising indicator of early progression to metastasis.

A biomarker of pro-metastatic PC, cancer that is likely to metastasise should be able to predict metastatic PC before progression and stratify aggressive PC from indolent PC early on. To further investigate PTN as a putative pro-metastatic marker, we assayed the tissue levels of PTN in a cohort of 234 men treated by radical prostatectomy at Stanford University^11^ (Fig. 1e). The cohort has a median 10-year follow-up data in which 58 patients developed biochemical relapse and 15 men progressed to lymph node or bone metastasis (Fig. 1e–g and Supplementary Table S4). We found that high levels of PTN in localised PC tissues were predictive for biochemical recurrence (Fig. 1e, *P* < 0.05). We assigned these patients to the corresponding CPGs using the Cambridge Prognostic Group calculator (http://cambridgeprognosticgroup.com) CPG5 men had poorer outcomes after surgery with a shorter time to biochemical recurrence, while none of the CPG1 patients relapsed during the 10 years follow-up (Supplementary Fig. S2, *P* < 0.0001). In agreement with our serum data, CPG5 patients expressed higher levels of PTN in prostatectomy specimens when compared to CPG1 patients (Fig. 1f, *P* < 0.05). High PTN protein levels also correlated with individual clinicopathological features including pre-operative serum PSA levels (*P* = 0.003), advanced pathological T stage (*P* = 0.0083) and higher Gleason grade groups (*P* = 0.0012) (Supplementary Tables S4 and  S5). Patients who later developed biochemical recurrence or metastasis also exhibited high levels of PTN in their prostatectomy specimens at the time of surgery when compared to patients who did not develop recurrence (Fig. 1g, *P* < 0.05). Multivariate analysis further showed that high levels of PTN was an independent predictor of biochemical recurrence in patients with otherwise favourable pathological Gleason grades 3 + 3 and 3 + 4 (Fig. 1h, *P* < 0.05). These results support the notion of PTN as an early marker of pro-metastatic disease and may be particularly relevant in men where other clinic-pathological features appear favourable.

## Discussion

In this study, our interest was driven by the goal of identifying biomarkers of lethal disease at the pre-dissemination stage. PTN was selected for further investigations as it was elevated in both aggressive non-metastatic as well as metastatic disease. PTN is a secreted growth factor with diverse functions related to tumour growth, angiogenesis and metastasis.^12–14^ PTN has been shown to play a functional role in PC growth and metastasis. It has been demonstrated that PTN level is regulated by androgens and androgen receptor during prostate development, and PTN regulates both mesenchymal and epithelial proliferation in prostatic cells.^15^ In several studies, overexpression of PTN results in a more aggressive phenotype, whereas downregulation of PTN expression leads to a decrease in growth, migration and invasion of PC cells in vitro and tumour growth and metastasis in vivo.^14,16–18^ In addition, a synthetic peptide that inhibits PTN function has been shown to delay PC cell and tumour growth in vitro and in vivo.^19,20^ Moreover, high levels of PTN were detected in the serum of patients with multiple cancer types, including breast cancer, lung cancer, myeloma and testicular cancer.^21–25^ High levels of serum PTN also correlate with poor survival in patients with small cell lung cancer (SCLC), and serve as a valuable diagnostic and prognostic biomarker for SCLC.^21^ A meta-analysis identified PTN as a promising biomarker for the prediction of unfavourable outcomes in cancer.^26^ In this context and to the best of our knowledge, PTN might be the first serum-based biomarker of “pro-metastatic” PC. Our study demonstrates that elevated PTN levels were observed in both serum and tissue of disease with locally advanced and high-grade features. It is possible that these high levels of PTN are due to micro-metastatic spread not visible on conventional imaging. Future studies incorporating PSMA-PET would be helpful to elucidate this point. PTN expression was also elevated in otherwise apparently good prognosis low/intermediate-grade disease, which went on to recur raising the potential for use as an early independent marker for adjuvant treatment. Taken together, these data support further research in larger independent cohorts to establish the veracity of our results and the utility of PTN as a clinical test.

## Supplementary information

Supplementary Figures and Tabels

## Data Availability

All data in this study are included as Supplementary Tables and Figures. Any additional information is available upon request.
