# Supplementary material for: Discovery of PTN as a serum-based biomarker of pro-metastatic prostate cancer
Source: Br J Cancer. 2020 Dec 8;124(5):896–900. doi: 10.1038/s41416-020-01200-0 (PMC7921397; doi:10.1038/s41416-020-01200-0)
Supplement: Supplementary file 1 — Supplementary Figures and Tabels [file 41416_2020_1200_MOESM1_ESM.pdf]

Supplementary Figure S1

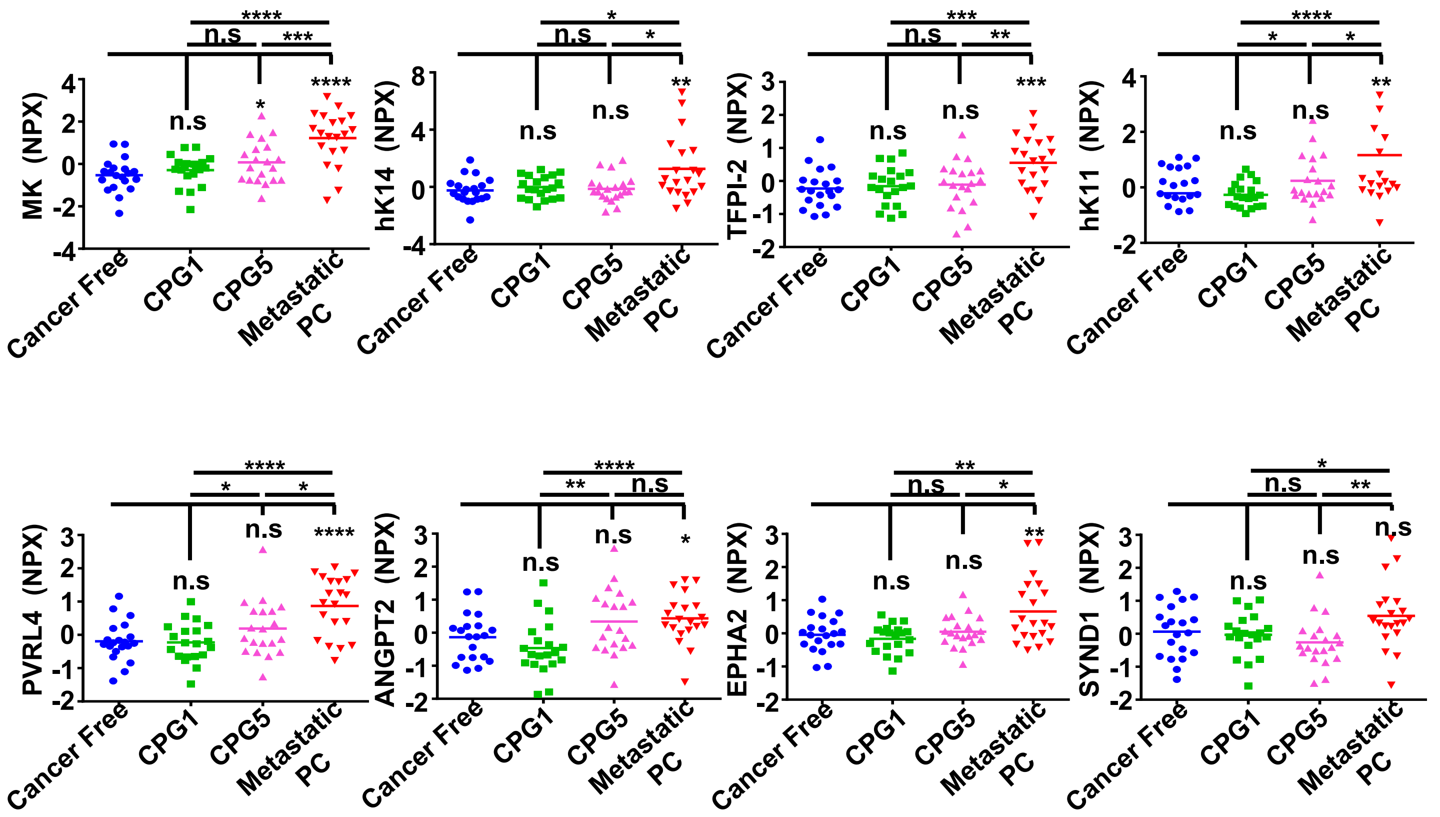

Figure S1. Candidate serum-based biomarkers for metastatic PC. Normalized protein levels for MK, PVRL4, EPHA2, TFPI-2, hK11, SYND1, ANGPT2, and hK14 is shown.

Supplementary Figure S2

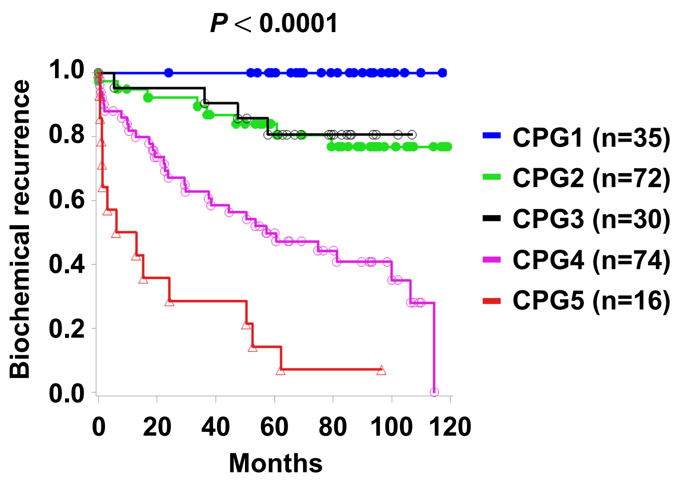

Figure S2. Outcome in reassigned CPGs from Stanford cohort. The Stanford cohort samples were assigned to appropriate CPGs by Cambridge Prognostic Group calculator. Biochemical recurrence status of CPGs was plotted.

Supplementary Table S1. Criteria of the Cambridge Prognostic Groups for discovering novel blood-based biomarkers for early detection of poor prognosis prostate cancer

Group	Criteria	Survival
Cancer Free	Absence of prostate cancer (systemic and image guided biopsy)	
CPG1	Gleason score 6 (Grade Group 1) AND PSA < 10 ng/ml AND Stages T1–T2	97% 10-year survival
CPG5	Any one of these criteria:<br>1. Gleason score 8 (Grade Group 4) + PSA > 20 ng/ml;<br>2. Gleason score 8 (Grade Group 4) + Stage T3;<br>3. Gleason score 9–10 (Grade Group 5);<br>4. Stage T4.	50% 10-year survival
Metastasis Group	With metastatic disease at diagnosis	

Supplementary Table S2. Summary of Olink biomarker panels

Panel	Biomarkers				
Immuno-Oncology Panel	ADA	CASP-8	IL-18	MCP-3	CD40-L
	ADGRG1	FasL	IL-2	MCP-4	CD28
	ANGPT1	FGF2	IL-33	MUC-16	TRAIL
	TIE2	CX3CL1	IL-4	NCR1	TWEAK
	ANGPT2	Gal-1	IL5	CD244	TNF
	ARG1	Gal-9	IL6	KLRD1	TNFSF14
	CCL17	GZMA	IL-7	NOS3	TNFRSF12A
	CCL19	GZMB	IL-8	PGF	TNFRSF21
	CCL20	GZMH	KIR3DL1	PDGF-B	TNFRSF4
	CCL23	HO-1	LAP TGFb1	PTN	TNFRSF9
	CCL3	HGF	LAG3	EGF	VEGF-A
	CCL4	ICOSLG	LAMP3	PD-L1	VEGFR-2
	CXCL1	IFN-gamma	CSF-1	PD-L2)	CD40
	CXCL10	IL-1 alpha	MMP-12	PDCD1	CD70
	CXCL11	IL10	MMP-7	CXCL12	CD83
	CXCL13	IL-12	MIC-A/B	CD4	CRTAM
	CXCL5	IL12RB1	MCP-1	CD5	DCN
	CXCL9	IL-13	MCP-2	CD8A	CD27
	CAIX	IL15			
Oncology Panel II	5'-NT	ADAM8	IFN-γ-R1	CYR61	TLR3
	ADAM-TS 15	ESM-1	IL6	S100A11	TGF-alpha
	TXLNA	EPHA2	hK11	S100A4	GPNMB
	AREG	FasL	KLK13	RET	TNFSF13
	ANXA1	FADD	hK14	RSPO3	TNFRSF19
	CD207	FCRLB	hK8	HER2	TNFRSF4
	CXCL13	FGF-BP1	LYPD3	HER3	TNFRSF6B
	CAIX	FR-alpha	MIA	HER4	ABL1
	CPE	FR-gamma	MSLN	SCAMP3	LYN
	CEA	FUR	MetAP 2	SEZ6L	VEGF-A
	CEACAM1	Gal-1	MIC-A/B	SPARC	VEGFR-2
	CTSV	GPC1	MK	SCF	VEGFR-3
	CD160	GZMB	MAD h5	SYND1	CXCL17
	CD27	GZMH	MUC-16	TCL1A	VIM
	CD48	HGF	PVRL4	LY9	WFDC2
	CD70	ICOSLG	PPY	TGFR-2	WIF-1
	CRNN	IGF1R	PODXL	TFPI-2	WISP-1
	CDKN1A	ITGAV	EGF	TRAIL	XPNPEP2
	DLL1	ITGB5			

Supplementary Table S3

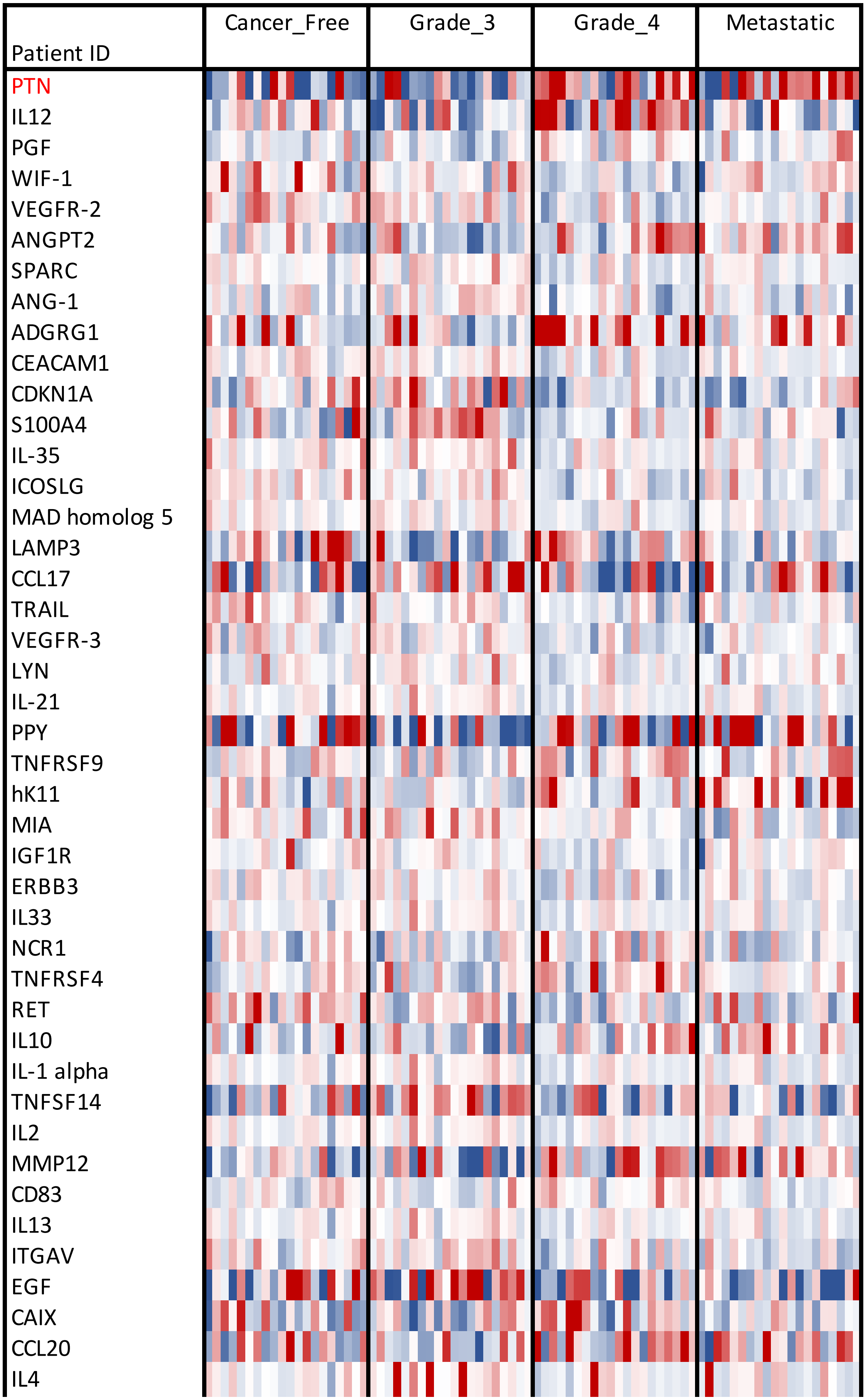

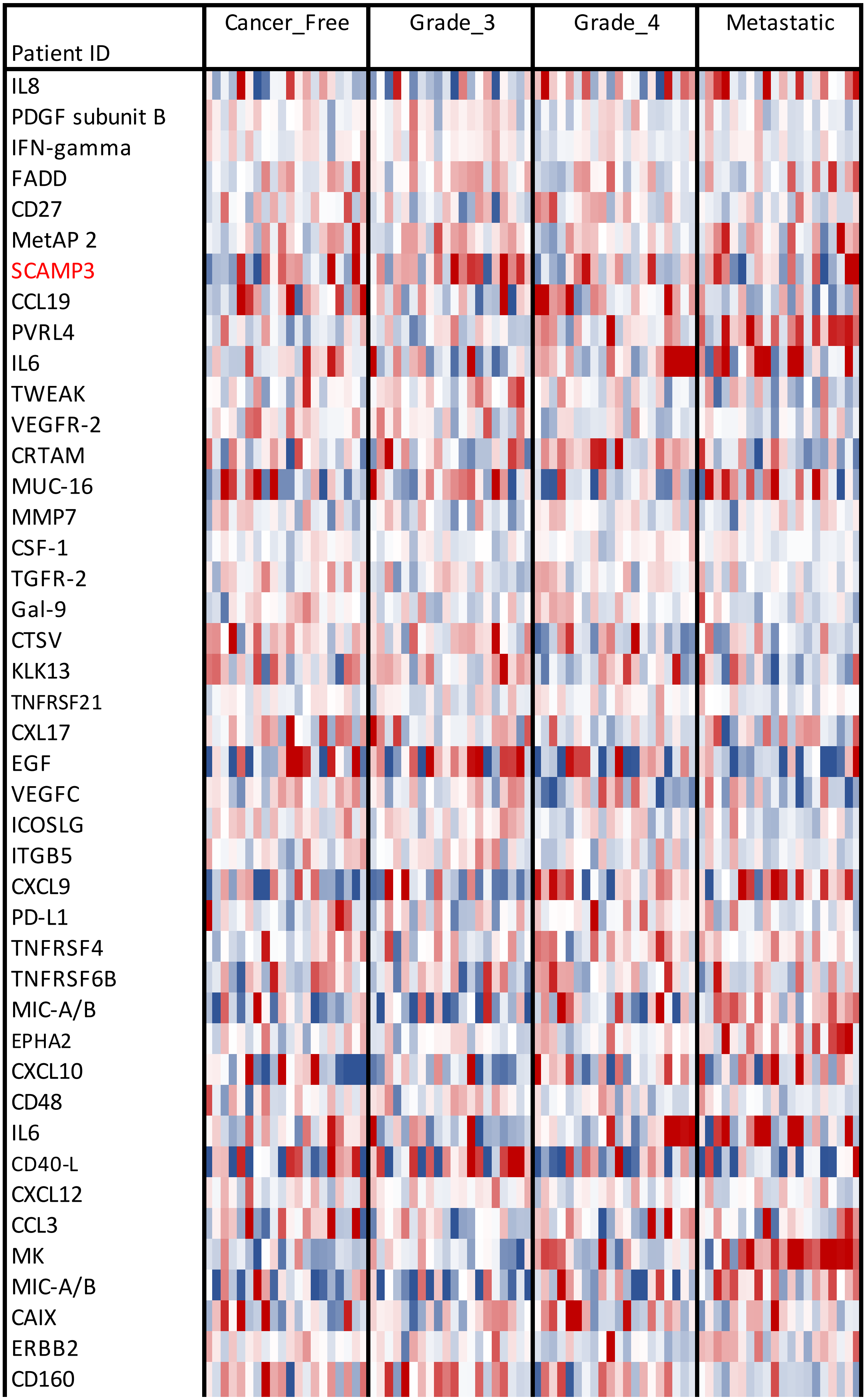
Patient ID
Cancer_Free
Grade_3
Grade_4
Metastatic
IL8
PDGF subunit B
IFN-gamma
FADD
CD27
MetAP 2
SCAMP3
CCL19
PVRL4
IL6
TWEAK
VEGFR-2
CRTAM
MUC-16
MMP7
CSF-1
TGFR-2
Gal-9
CTSV
KLK13
TNFRSF21
CXL17
EGF
VEGFC
ICOSLG
ITGB5
CXCL9
PD-L1
TNFRSF4
TNFRSF6B
MIC-A/B
EPHA2
CXCL10
CD48
IL6
CD40-L
CXCL12
CCL3
MK
MIC-A/B
CAIX
ERBB2
CD160

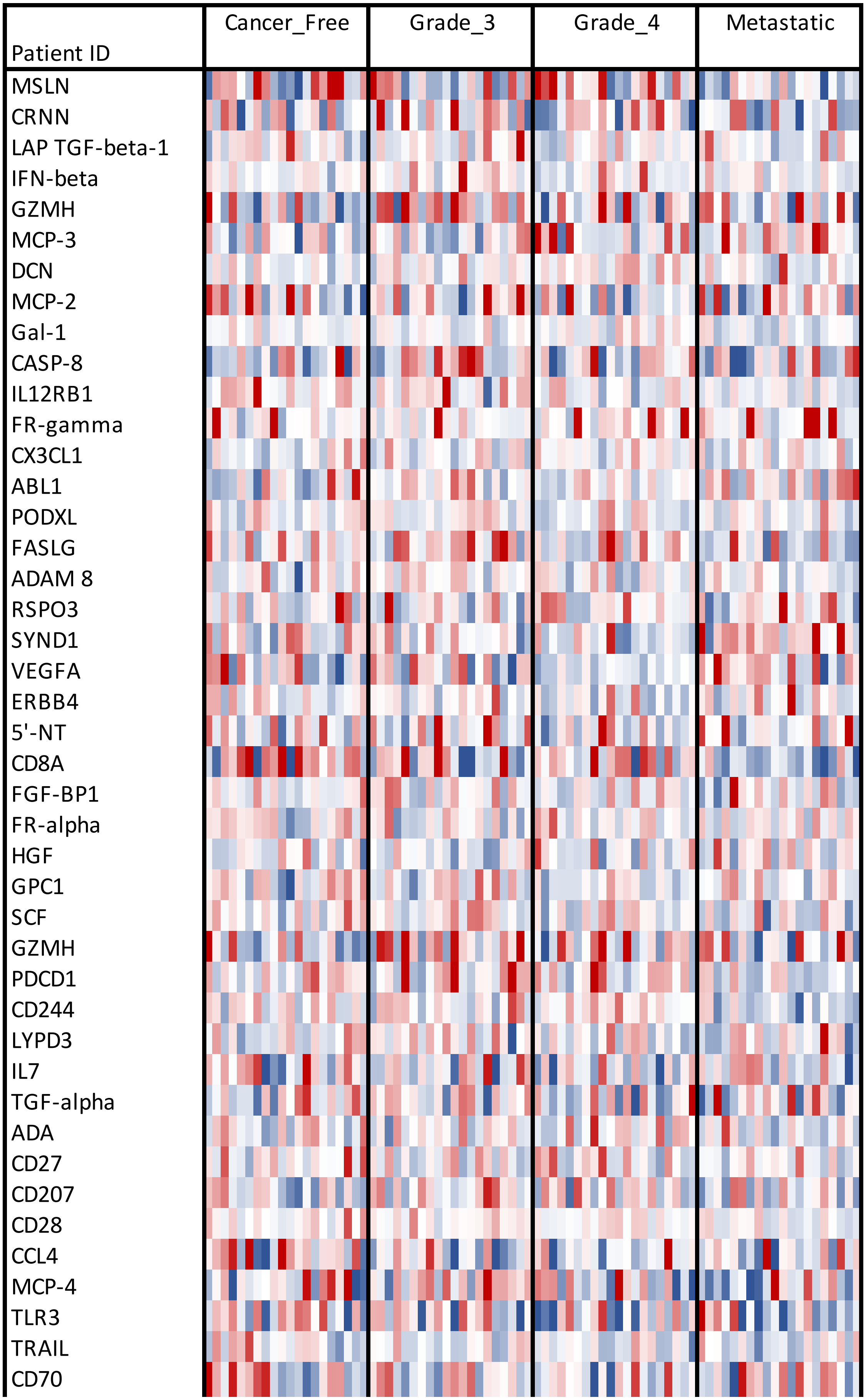
Patient ID
Cancer_Free
Grade_3
Grade_4
Metastatic
MSLN
CRNN
LAP TGF-beta-1
IFN-beta
GZMH
MCP-3
DCN
MCP-2
Gal-1
CASP-8
IL12RB1
FR-gamma
CX3CL1
ABL1
PODXL
FASLG
ADAM 8
RSPO3
SYND1
VEGFA
ERBB4
5'-NT
CD8A
FGF-BP1
FR-alpha
HGF
GPC1
SCF
GZMH
PDCD1
CD244
LYPD3
IL7
TGF-alpha
ADA
CD27
CD207
CD28
CCL4
MCP-4
TLR3
TRAIL
CD70

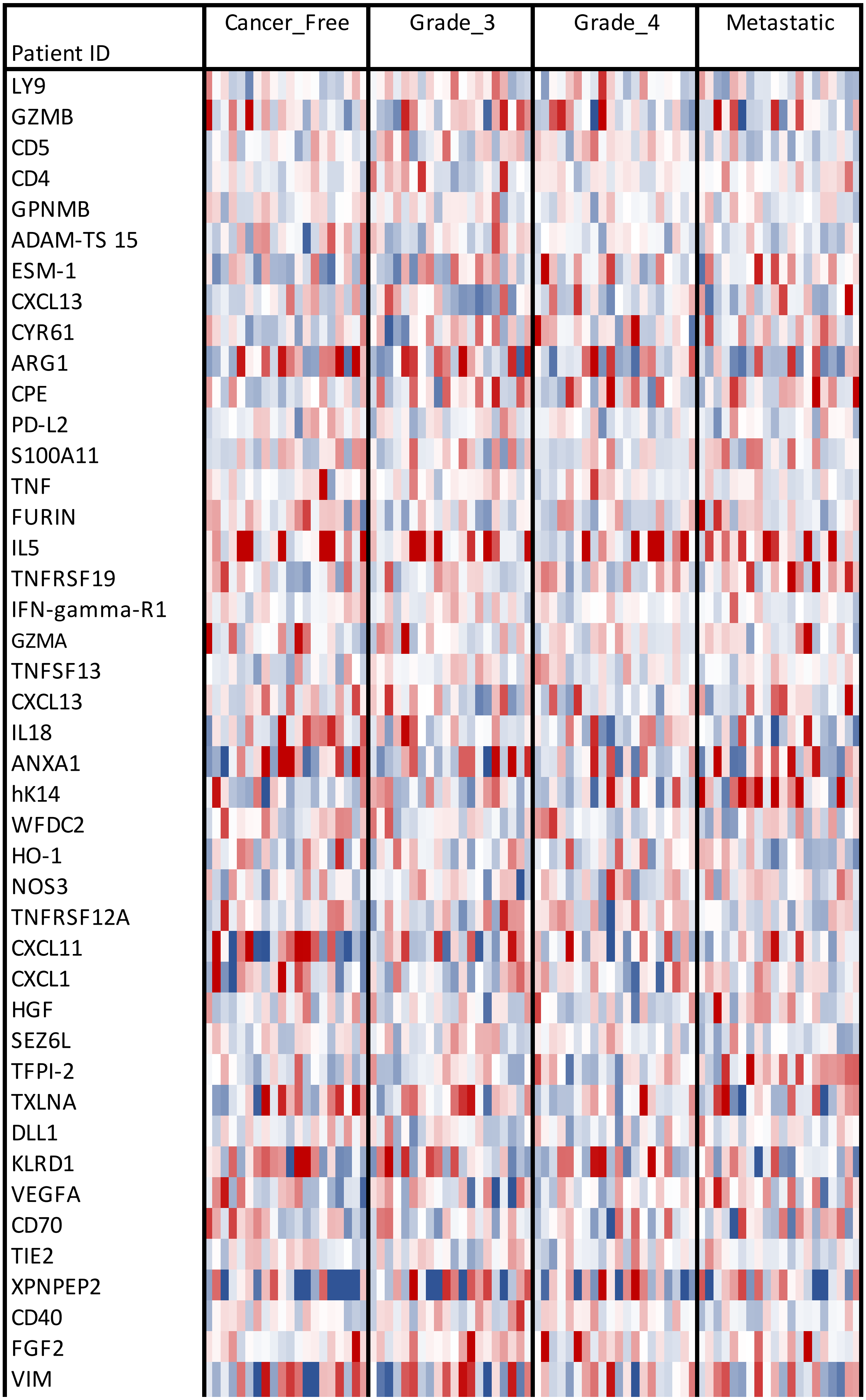
Patient ID
Cancer_Free
Grade_3
Grade_4
Metastatic
LY9
GZMB
CD5
CD4
GPNMB
ADAM-TS 15
ESM-1
CXCL13
CYR61
ARG1
CPE
PD-L2
S100A11
TNF
FURIN
IL5
TNFRSF19
IFN-gamma-R1
GZMA
TNFSF13
CXCL13
IL18
ANXA1
hK14
WFDC2
HO-1
NOS3
TNFRSF12A
CXCL11
CXCL1
HGF
SEZ6L
TFPI-2
TXLNA
DLL1
KLRD1
VEGFA
CD70
TIE2
XPNPEP2
CD40
FGF2
VIM

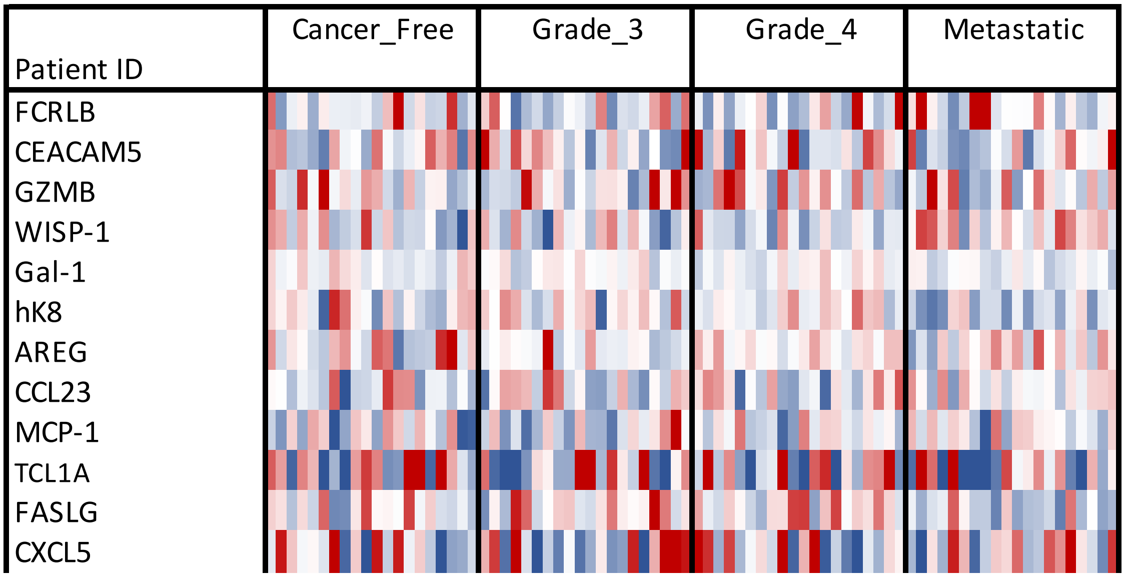
Patient ID
Cancer_Free
Grade_3
Grade_4
Metastatic
FCRLB
CEACAM5
GZMB
WISP-1
Gal-1
hK8
AREG
CCL23
MCP-1
TCL1A
FASLG
CXCL5

Supplementary Table S4. Summary of Stanford study cohort (N = 234)

Baseline characteristic	Study population<br>N = 227*	PTN<br>Negative/Low<br>N = 102	PTN<br>Medium/High<br>N = 125	P value
CPG, N (%)	CPG1 35 (15%)<br>CPG5 16 (7%)	CPG1 21 (60%)<br>CPG5 4 (25%)	CPG1 14 (40%)<br>CPG5 12 (75%)	**0.034**
Age at baseline, years, median (IQR)	65 (60.8-68.5)	65 (60.6-68.6)	65 (60.9-68.5)	0.81
PSA, µg/L, median (IQR)	7.7 (5.1-12.8)	6.9 (4.3-10.9)	8.0 (5.8-14.5)	**0.003**
Pathological Grade, N (%)				**0.0012**
≤ 6	46 (20%)	26 (25%)	20 (16%)	
3 + 4	132 (58%)	55 (54%)	77 (62%)	
4 + 3	47 (21%)	21 (21%)	26 (21%)	
8-10	2 (1%)	0 (0%)	2 (2%)	
Pathological T-stage, N (%)				**0.0083**
pT2	146 (64%)	71 (70%)	75 (60%)	
pT3	78 (34%)	31 (30%)	47 (38%)	
pT4	3 (1%)	0 (0%)	3 (2%)	
N-stage, N (%)				0.242
N0/x	216 (95%)	97 (95%)	119 (95%)	
N1	11 (5%)	5 (5%)	6 (5%)	
Margin status, N (%)$^{+}$				0.335
R-	182 (81%)	85 (83%)	97 (78%)	
R+	44 (19%)	17 (17%)	27 (22%)	

*Seven samples were excluded from the cohort due to lack of sufficient tissue.
P value: Mann-Whitney U test for continuous variables, X^2 test for categorical variables and Fisher's exact test for CPG, pathological grade, pathological T-stage, N-stage and margin status.
PSA, prostate specific antigen.

Supplementary Table S5

ID	Preop PSA (ng/ml)	Path T	Path Grade	PTN levels
1	13.40	T3a	3+4	Negative/Low
2	29.70	T3b	4+3	Negative/Low
3	11.70	T3b	4+3	Negative/Low
4	12.60	T3a	3+4	Negative/Low
5	22.06	T2b	3+3	Negative/Low
6	5.61	T3a	3+4	Negative/Low
7	19.60	T3a	4+3	Negative/Low
8	9.29	T2b	3+4	Negative/Low
9	79.20	T4	3+4	Negative/Low
10	8.19	T2b	3+4	Negative/Low
11	27.00	T2b	3+4	Negative/Low
12	64.35	T4	4+3	Negative/Low
13	15.07	T3b	4+3	Negative/Low
14	7.80	T3b	3+4	Negative/Low
15	35.00	T3a	3+4	Negative/Low
16	17.80	T3a	3+4	Negative/Low
17	15.73	T2	3+3	Negative/Low
18	4.18	T2b	3+3	Negative/Low
19	3.41	T3b	4+3	Negative/Low
20	5.17	T3a	3+4	Negative/Low
21	6.10	T3a	4+3	Negative/Low
22	3.74	T2b	3+4	Negative/Low
23	6.98	T3a	3+4	Negative/Low
24	11.00	T3a	3+4	Negative/Low
25	12.20	T3a	3+4	Negative/Low
26	8.00	T2b	4+3	Negative/Low
27	5.10	T2b	3+3	Negative/Low
28	5.17	T2b	3+4	Negative/Low
29	5.30	T3a	4+3	Negative/Low
30	1.70	T2b	3+3	Negative/Low
31	21.45	T3b	3+4	Negative/Low
32	11.50	T2b	3+4	Negative/Low
33	2.47	T2b	3+4	Negative/Low
34	6.43	T2b	3+3	Negative/Low
35	5.40	T2b	3+4	Negative/Low
36	41.70	T3b	4+5	Negative/Low
37	5.80	T3a	3+4	Negative/Low
38	10.00	T2b	3+4	Negative/Low
39	15.34	T2b	3+4	Negative/Low
40	7.20	T2b	3+4	Negative/Low
41	24.97	T3b	3+4	Negative/Low
42	3.60	T2b	3+4	Negative/Low
43	6.80	T2b	3+4	Negative/Low
44	18.00	T3a	4+3	Negative/Low

45	2.86	T2b	3+3	Negative/Low
46	4.65	T2a	3+4	Negative/Low
47	7.80	T2b	3+3	Negative/Low
48	13.90	T3a	3+4	Negative/Low
49	1.15	T2b	3+4	Negative/Low
50	4.40	T3a	3+4	Negative/Low
51	1.59	T2a	3+4	Negative/Low
52	1.70	T2b	3+4	Negative/Low
53	19.90	T3a	4+3	Negative/Low
54	3.40	T2b	3+3	Negative/Low
55	35.47	T3b	4+3	Negative/Low
56	2.31	T2b	3+3	Negative/Low
57	11.00	T2b	3+4	Negative/Low
58	5.50	T3a	4+3	Negative/Low
59	14.90	T3a	3+4	Negative/Low
60	4.60	T3a	3+4	Negative/Low
61	20.90	T2b	3+4	Negative/Low
62	5.83	T3a	4+3	Negative/Low
63	5.72	T3a	3+4	Negative/Low
64	17.90	T2	3+4	Negative/Low
65	9.79	T2	4+3	Negative/Low
66	5.06	T3a	3+4	Negative/Low
67	10.89	T2b	3+4	Negative/Low
68	7.37	T2b	3+4	Negative/Low
69	7.26	T3a	3+4	Negative/Low
70	4.07	T2b	3+4	Negative/Low
71	62.50	T3a	4+3	Negative/Low
72	36.63	T3a	3+4	Negative/Low
73	29.30	T2b	3+4	N/A Lack of tissue
74	9.30	T2b	3+4	Negative/Low
75	5.40	T3a	4+3	Negative/Low
76	6.93	T2b	3+4	N/A Lack of tissue
77	12.30	T2b	3+4	Negative/Low
78	13.10	T2b	3+3	Negative/Low
79	11.90	T4	3+4	Negative/Low
80	30.25	T2a	4+3	Negative/Low
81	6.90	T3a	3+4	Negative/Low
82	62.70	T3a	4+3	Negative/Low
83	16.20	T2b	3+3	Negative/Low
84	7.80	T3a	3+4	Negative/Low
85	12.20	T3a	3+4	Negative/Low
86	7.60	T3b	4+3	Negative/Low
87	10.90	T2b	3+4	Negative/Low
88	19.60	T3b	3+4	Negative/Low
89	13.86	T3a	3+4	Negative/Low

90	22.00	T3b	4+3	Negative/Low
91	3.63	T2b	3+4	Negative/Low
92	13.30	T3a	3+4	Negative/Low
93	41.40	T3a	3+3	N/A Lack of tissue
94	2.30	T2	3+4	Negative/Low
95	12.70	T2b	3+4	Negative/Low
96	2.47	T2b	3+3	Negative/Low
97	10.45	T3a	3+3	Negative/Low
98	6.70	T3a	3+4	Negative/Low
99	7.09	T3a	3+4	Negative/Low
100	13.30	T3a	3+4	Negative/Low
101	1.00	T3a	3+4	Negative/Low
102	4.17	T2b	3+3	Negative/Low
103	16.80	T3a	4+3	Negative/Low
104	7.86	T3a	3+4	Negative/Low
105	3.18	T2b	3+3	Negative/Low
106	3.90	T2b	3+4	Medium/High
107	8.03	T2b	3+4	Medium/High
108	4.19	T2b	3+4	Medium/High
109	1.87	T2	3+3	Medium/High
110	12.98	T2b	3+4	Medium/High
111	2.34	T2a	3+4	Medium/High
112	27.90	T2b	3+4	Medium/High
113	1.70	T3a	3+4	Medium/High
114	9.00	T2a	3+4	Medium/High
115	16.00	T3b	4+3	Medium/High
116	9.50	T3a	4+3	Medium/High
117	4.40	T2b	3+4	Medium/High
118	4.77	T2a	3+4	Medium/High
119	6.90	T3a	3+4	Medium/High
120	7.81	T3a	4+3	Medium/High
121	6.10	T2b	3+4	Medium/High
122	3.56	T2b	3+4	N/A Lack of tissue
123	2.18	T2b	3+4	Medium/High
124	10.05	T2b	3+4	Medium/High
125	6.88	T2b	3+4	Medium/High
126	5.79	T2b	3+4	N/A Lack of tissue
127	6.00	T2b	3+2	Medium/High
128	2.15	T2b	3+4	Medium/High
129	7.24	T2b	3+4	Medium/High
130	6.71	T2b	3+4	Medium/High
131	6.29	T2b	3+3	Medium/High
132	5.01	T2b	3+3	Medium/High
133	7.57	T2b	4+3	Medium/High
134	3.22	T2	3+3	Medium/High

135	7.94	T2	3+4	Medium/High
136	13.75	T3b	4+3	Medium/High
137	15.87	T2b	3+4	Medium/High
138	7.90	T2a	4+3	Medium/High
139	4.05	T2b	4+3	Medium/High
140	6.52	T2a	3+4	Medium/High
141	6.16	T2b	3+4	Medium/High
142	9.04	T2a	3+4	Medium/High
143	9.10	T3a	3+4	Medium/High
144	5.75	T2b	3+3	Medium/High
145	6.30	T2b	3+4	Medium/High
146	18.23	T2b	3+4	Medium/High
147	2.49	T2b	3+4	Medium/High
148	7.13	T3a	3+4	Medium/High
149	3.93	T2a	3+4	Medium/High
150	2.99	T2b	3+4	Medium/High
151	17.86	T2b	3+3	Medium/High
152	11.51	T2	3+4	Medium/High
153	8.70	T2b	3+4	Medium/High
154	3.69	T2b	3+4	Medium/High
155	3.84	T2b	3+4	Medium/High
156	50.39	T3a	4+3	Medium/High
157	4.07	T2b	3+3	Medium/High
158	8.27	T2b	3+4	Medium/High
159	17.05	T2b	4+3	Medium/High
160	7.40	T2b	3+4	Medium/High
161	22.30	T2a	3+3	Medium/High
162	5.99	T2a	3+4	Medium/High
163	10.52	T2b	3+4	Medium/High
164	8.82	T2b	3+4	Medium/High
165	8.07	T2b	4+3	Medium/High
166	7.01	T2b	3+4	Medium/High
167	9.78	T2b	4+3	Medium/High
168	8.04	T2a	3+4	Medium/High
169	2.21	T2b	3+3	Medium/High
170	0.84	T2b	3+3	Medium/High
171	17.17	T2b	3+4	Medium/High
172	36.78	T3a	4+3	Medium/High
173	3.85	T2b	3+4	Medium/High
174	12.28	T3b	3+4	Medium/High
175	6.87	T3a	3+4	Medium/High
176	7.47	T2b	3+4	Medium/High
177	5.42	T2b	3+3	Medium/High
178	6.60	T2a	3+3	Medium/High
179	19.16	T3a	4+3	Medium/High

180	14.19	T2b	3+4	Medium/High
181	6.00	T2b	3+3	Medium/High
182	4.40	T2b	3+3	Medium/High
183	1.70	T2b	3+3	Medium/High
184	3.52	T3a	4+3	Medium/High
185	25.99	T2b	3+3	Medium/High
186	15.92	T2b	4+3	Medium/High
187	4.60	T2b	3+4	Medium/High
188	4.50	T2b	3+3	Medium/High
189	7.41	T2b	3+4	Medium/High
190	10.79	T2b	3+4	Medium/High
191	9.66	T2b	3+4	Medium/High
192	7.79	T3a	3+4	Medium/High
193	14.86	T2b	3+4	Medium/High
194	11.20	T2b	3+3	Medium/High
195	5.71	T3a	3+4	N/A Lack of tissue
196	10.40	T2b	4+3	Medium/High
197	3.51	T3a	3+4	Medium/High
198	10.29	T2b	3+4	Medium/High
199	7.00	T2b	4+3	Medium/High
200	10.94	T3a	4+3	Medium/High
201	3.83	T2b	3+3	Medium/High
202	9.85	T2b	4+3	Medium/High
203	7.42	T3a	4+3	Medium/High
204	13.50	T2	4+3	Medium/High
205	7.97	T2b	3+3	Medium/High
206	5.13	T2b	3+4	Medium/High
207	12.02	T2b	3+4	Medium/High
208	8.66	T2a	3+4	Medium/High
209	7.22	T3a	4+3	Medium/High
210	6.11	T2b	3+4	Medium/High
211	5.54	T2b	3+3	Medium/High
212	9.00	T2b	3+3	Medium/High
213	6.94	T3b	4+3	Medium/High
214	8.20	T3a	3+4	Medium/High
215	30.56	T2b	3+4	Medium/High
216	4.90	T2b	3+3	Medium/High
217	11.44	T2b	3+4	N/A Lack of tissue
218	9.71	T2b	3+4	Medium/High
219	4.96	T3a	3+4	Medium/High
220	10.80	T2b	3+3	Medium/High
221	6.40	T3a	3+3	Medium/High
222	5.36	T2b	3+3	Medium/High
223	4.80	T2b	3+3	Medium/High
224	6.14	T3a	3+3	Medium/High

225	5.02	T2b	3+4	Medium/High
226	6.47	T2b	3+4	Medium/High
227	11.07	T2b	3+4	Medium/High
228	23.92	T2b	3+4	Medium/High
229	4.20	T2b	3+3	Medium/High
230	5.50	T2b	3+4	Medium/High
231	6.10	T2b	3+4	Medium/High
232	7.79	T2b	4+3	Medium/High
233	15.00	T3a	4+3	Medium/High
234	14.19	T3b	4+4	Medium/High

ID	Sample Stage	Sample Type	PSA Diagnosis	PSA Diagnosis Date
1	Benign	Serum	3.41	16/10/2014
2	Benign	Serum	9.1	01/11/2014
3	Benign	Serum	9.74	01/12/2016
4	Benign	Serum	4.3	19/01/2017
5	Benign	Serum	10.53	02/10/2017
6	Benign	Serum	12.27	22/09/2017
7	Benign	Serum	4.78	26/09/2017
8	Benign	Serum	7.7	05/10/2017
9	Benign	Serum	16.22	15/11/2017
10	Benign	Serum	4.93	08/11/2017
11	Benign	Serum	10.76	26/09/2017
12	Benign	Serum	5.8	16/01/2018
13	Benign	Serum	11.53	24/10/2014
14	Benign	Serum	7.68	17/11/2017
15	Benign	Serum	6.38	19/12/2017
16	Benign	Serum	62.02	22/11/2017
17	Benign	Serum	5.87	27/02/2018
18	Benign	Serum	7.34	22/03/2018
19	Benign	Serum	9.5	15/05/2017
20	Benign	Serum	10.78	29/12/2017
21	CPG1	Serum	7.7	01/12/2016
22	CPG1	Serum	4.8	01/12/2016
23	CPG1	Serum	3.4	22/09/2015
24	CPG1	Serum	9.4	02/04/2016
25	CPG1	Serum	3.26	17/01/2016
26	CPG1	Serum	2.4	11/06/2012
27	CPG1	Serum	5.53	09/11/2017
28	CPG1	Serum	11.49	09/11/2017
29	CPG1	Serum	6.9	22/01/2017
30	CPG1	Serum	7.02	12/10/2017
31	CPG1	Serum	4.27	12/10/2017
32	CPG1	Serum	2.92	26/10/2015
33	CPG1	Serum	6	15/06/2012
34	CPG1	Serum	6.77	24/11/2017
35	CPG1	Serum	3.3	14/10/2008
36	CPG1	Serum	5.29	08/07/2013
37	CPG1	Serum	2.91	13/10/2014
38	CPG1	Serum	5.8	04/04/2013
39	CPG1	Serum	3.62	09/12/2016
40	CPG1	Serum	7.1	02/09/2012
41	CPG5	Serum	68.43	18/10/2016
42	CPG5	Serum	123.33	31/10/2016
43	CPG5	Serum	122.57	02/11/2016
44	CPG5	Serum	10.73	9/6/16

45	CPG5	Serum	301.14	07/11/2016
46	CPG5	Serum	21.67	27/03/2017
47	CPG5	Serum	33.87	05/05/2017
48	CPG5	Serum	11.82	23/11/2017
49	CPG5	Serum	4.31	09/10/2017
50	CPG5	Serum	54	27/11/2017
51	CPG5	Serum	6.8	11/8/17
52	CPG5	Serum	19.3	03/07/2017
53	CPG5	Serum	50.65	1/16/18
54	CPG5	Serum	20.29	16/02/2018
55	CPG5	Serum	8.42	10/12/17
56	CPG5	Serum	11	19/06/2017
57	CPG5	Serum	32.33	07/08/2017
58	CPG5	Serum	12.83	15/11/2017
59	CPG5	Serum	6.68	05/01/2018
60	CPG5	Serum	13	04/12/2017
61	Metastatic	serum	55.49	22/10/2014
62	Metastatic	serum	11.39	23/02/2015
63	Metastatic	serum	128.83	06/03/2015
64	Metastatic	serum	58.47	10/11/2014
65	Metastatic	serum	99.06	03/11/2014
66	Metastatic	serum	10.3	20/01/2015
67	Metastatic	serum	100	28/01/2015
68	Metastatic	serum	75.52	21/04/2015
69	Metastatic	serum	130.2	09/06/2015
70	Metastatic	serum	29.81	30/04/2015
71	Metastatic	serum	10.1	29/06/2015
72	Metastatic	serum	96.74	17/06/2015
73	Metastatic	serum	270.25	10/10/2016
74	Metastatic	serum	222.93	05/07/2017
75	Metastatic	serum	355.55	05/03/2018
76	Metastatic	serum	230.04	03/10/2017
77	Metastatic	serum	1255.12	22/02/2018
78	Metastatic	serum	193.4	02/02/2018
79	Metastatic	serum	844.74	28/03/2018
80	Metastatic	Serum	7255.83	16/04/2018
